# Body mass index affects proliferation and osteogenic differentiation of human subcutaneous adipose tissue-derived stem cells

**DOI:** 10.1186/1471-2121-14-34

**Published:** 2013-08-07

**Authors:** Trivia P Frazier, Jeffrey M Gimble, Jessica W Devay, Hugh A Tucker, Ernest S Chiu, Brian G Rowan

**Affiliations:** 1 Department of Structural and Cellular Biology, Tulane University, New Orleans, LA, USA; 2 Stem Cell Biology Laboratory, Pennington Biomedical Research Center, Louisiana State University System, Baton Rouge, LA, USA; 3 Stem Cell Core Facility, Tulane University, New Orleans, LA, USA; 4 Flow Cytometry Core Facility, Center for Gene Therapy, Tulane University, New Orleans, LA, USA; 5 Division of Plastic & Reconstructive Surgery, Louisiana State University Health Science Center, New Orleans, LA, USA

**Keywords:** Adipose stromal/stem cells (ASCs), Body mass index (BMI), Osteogenesis, Proliferation, Colony formation, Cell size

## Abstract

**Background:**

Obesity is associated with a higher risk of developing cancer and co-morbidities that are part of the metabolic syndrome. Adipose tissue is recognized as an endocrine organ, as it affects a number of physiological functions, and contains adipose tissue-derived stem cells (ASCs). ASCs can differentiate into cells of multiple lineages, and as such are applicable to tissue engineering and regenerative medicine. Yet the question of whether ASC functionality is affected by the donor’s body mass index (BMI) still exists.

**Results:**

ASCs were isolated from patients having different BMIs (BMI-ASCs), within the ranges of 18.5-32.8. It was hypothesized that overweight BMI-ASCs would be more compromised in early adipogenic and osteogenic potential, and ability to form colonies *in vitro*. BMI was inversely correlated with ASC proliferation and colony forming potential as assessed by CyQUANT proliferation assay (fluorescence- based measurement of cellular DNA content), and colony forming assays. BMI was positively correlated with early time point (day 7) but not later time point (day 15) intracytoplasmic lipid accumulation as assessed by Oil-Red-O staining. Alizarin red staining and RT-PCR for alkaline phosphatase demonstrated that elevated BMI resulted in compromised ASC mineralization of extracellular matrix and decreased alkaline phosphatase mRNA expression.

**Conclusions:**

These data demonstrate that elevated BMI resulted in reduced ASC proliferation, and potentially compromised osteogenic capacity *in vitro*; thus BMI is an important criterion to consider in selecting ASC donors for clinical applications.

## Background

The increasing epidemic of obesity within the United States has been associated with a higher risk of developing co-morbidities that are categorized as part of the metabolic syndrome that include dyslipoproteinemia (raised triglyceride and/or reduced high density lipoprotein cholesterol levels), diabetes mellitus, and cardiovascular and coronary artery diseases. Obesity has also been identified as a risk factor for an increased incidence of several forms of cancer, including colon and breast cancers [1-5]. A high incidence of these morbidities exists in obese individuals, both adolescents and adults [3,4,6]. Recent studies have also investigated the effects of obesity on the musculoskeletal system [7], and have identified obesity as an independent risk factor for increased bone fracture risk [8] and clinical implant failure following total joint replacement [9-14]. Obese individuals with higher body mass indices (BMIs; the ratio of body weight (kg) to height (m^2^)) exhibit lower relative bone area and bone mass compared to non-obese individuals [15]. While various explanations have been widely suggested, our knowledge relating the pathogenesis of obesity at the cellular level and its potential impact in tissue engineering and regenerative medicine applications is very limited.

Adipose tissue is a complex, highly active endocrine organ that secretes bioactive peptides, or adipocytokines, that are known to affect a number of physiological functions in the reproductive system, neuroendocrine system, rennin-angiotensin system, and in bone metabolism. [16-18]. Adipose tissue contains mature adipocytes, endothelial cells, cells of the immune system, and a small percentage of the adipocyte precursors termed adipose tissue-derived stem cells (ASCs). The ability of ASCs to differentiate into cells of the endodermal, mesodermal, and ectodermal lineages makes ASCs optimal candidates for applications in cellular therapies [19], including tissue engineering and regenerative medicine [20-23]. These applications potentially involve the repair of the musculoskeletal and other biological systems [24,25]. However, the growing interest in ASCs for cell therapeutics has led to questions about donor physiological conditions on ASC functionality (i.e. effects on viability, differentiation, and growth properties).

Recent reports have positively correlated BMI with ASC yields from adipose tissue and inversely correlated BMI with adipocyte size [26]. BMI has also been inversely correlated with bone-marrow derived mesenchymal stem cell (BMSC) cyclic tensile strain capacity, or mechano-response, and alkaline phosphatase activity *in vitro,* which suggested a possible compromise in the osteogenic differentiation potential of BMSCs from individuals with higher BMIs [5]. Similar studies on ASCs have not been conducted.

The current study investigated the proliferation ability, *in vitro* differentiation potential, relative cell volume and complexity, and colony forming potential of ASCs isolated from patients having different BMIs, within the ranges of 18.5-24.9 kg/m^2^, (designated lean BMI-ASCs), and 25–32.8 kg/m^2^, (designated overweight BMI-ASCs). It was hypothesized that overweight BMI-ASCs would be more compromised in the ability to proliferate, differentiate, and form colonies *in vitro,* thereby contributing to the problematic obesity-associated pathologies, and therefore BMI should be considered when using ASCs for regenerative medicine applications.

## Results

### Percent serum and BMI inversely correlated with ASC growth

**Figure 1:**
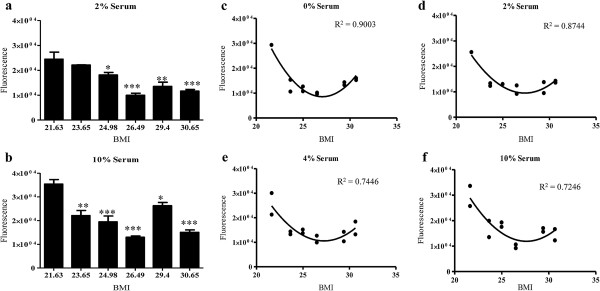
**Higher BMI-ASCs exhibited compromised growth when exposed to low serum *in vitro*. a**, **b**. CyQUANT was performed following ASC exposure to low serum (0-10% FBS supplementation) culture conditions for 48 hrs. Percentage serum and BMI both significantly affect ASC growth. **c**-**f**. Regression analysis of BMI-ASC growth when exposed to low serum *in vitro*. Nonlinear regression analysis performed using least squares ordinary fit. R^2^ values indicate a strong correlation between BMI, percent serum exposure, and ASC growth. Values are reported as N ± SE; * p > 0.05, ** p > 0.001, *** p > 0.0001.

Cryopreserved ASCs isolated from lipoaspirates of women with different body mass indices (BMIs; Table 1), were cultured for up to 72 hours in ASC culture medium supplemented with 0 to 10% FBS. Cell growth was measured by MTT and CyQUANT cell proliferation assays. Growth data reflected an inverse relationship between BMI and ASC growth *in vitro* (Figure 1a,b). The largest effect was observed in 2% serum for 48 hrs (Figure 1a); however, growth was also compromised when ASCs were cultured 10% serum (Figure 1b). MTT data also revealed a time-dependent biphasic response in cell growth in which full recovery and maximum growth occurred at 72 hrs following a decline in higher BMI-ASC growth at 24 hrs and 48 hrs (data not shown). Non linear regression analyses using least fit ordinary squares supported the strong inverse relationship between both BMI (determination coefficient, R^2^ = 0.90; p < 0.05) and serum (R^2^ = 0.86; p < 0.05) on ASC growth (Figure 1c-f), where culture in the lowest percent serum (0%) reflected the strongest determination coefficient. Quadratic equations were used for nonlinear regression analyses, curve-fitting and subsequent R^2^ values (reported in Figure 1c-f).

**Table 1:** BMI-ASCs used in the study

	**Lot number**	**Age**	**Body mass index**
**BMI < 25**	L080125	47	19.99
	L090514	40	21.18
	L070430	33	21.63
	L080508	42	21.97
	L080211	48	23.65
	L080401	44	24.98
	Mean ± SD	42.3 ± 5.46	22.2 ± 1.79
**BMI > 25**	L071025	61	26.49
	L070918	33	29.4
	L070525	37	30.65
	L101129W	44	30.94
	L100928W	28	31.58
	L100412W	41	32.75
	Mean ± SD	42.3 ± 5.46	22.2 ± 1.79
**All donors**	Mean ± SD	42.3 ± 5.46	22.2 ± 1.79

All ASCs were obtained from subcutaneous abdominal adipose tissue of female, Caucasian donors of ages between 28 and 61 with a mean ± SD of 41.5 ± 8.61 years. ASCs isolated from patients having different body mass indices (BMIs) were designated BMI-ASCs. BMI-ASCs used in the study were grouped into categories and identified as either ‘lean BMI-ASCs’ (BMI <25) or ‘overweight BMI-ASCs’ (>30). The patients displayed a mean body mass index (kg/m^2^) (±SD) of 26.3 ± 4.62.

### BMI negatively correlated with colony-forming unit potential

**Figure 2:**
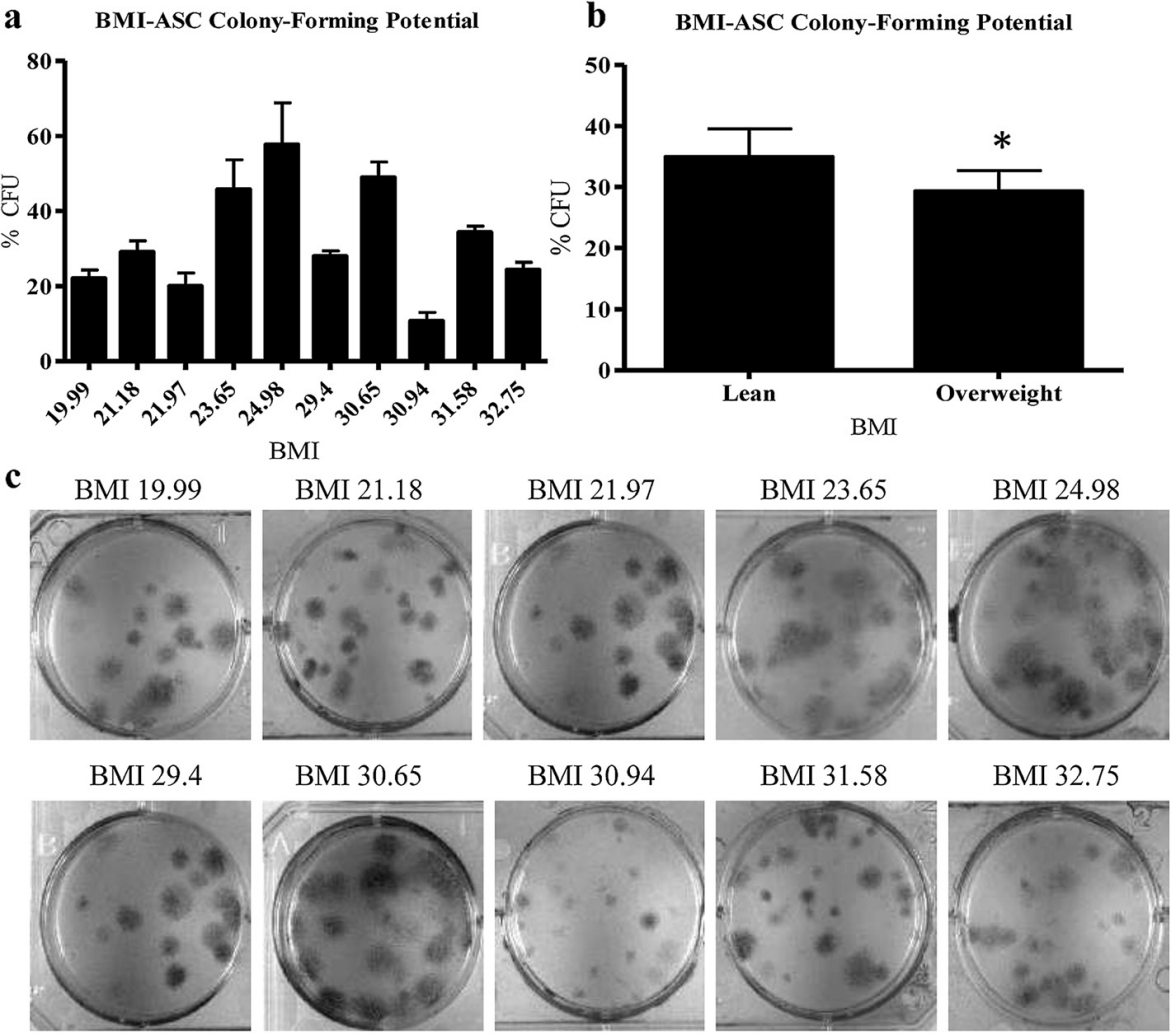
**BMI negatively correlated with ASC colony-forming potential *in vitro.*** ASCs were plated at a concentration of 100 cells/mL in 6 well plates and cultured for 14 days. The number of colonies per plate divided by the cells plated x 100 was determined as the “% CFU”. **a**. Ungrouped BMI-ASC colony-forming potential, and **b**. grouped BMI-ASC CFU potential. **c**. Visualization of colonies formed in 100 cm^2^ dishes. Values reported as N ± SE; * p > 0.05.

To further examine differences in BMI-ASC growth, colony-forming unit (CFU) assays were performed on the BMI-ASC donors (Table 1). These donors were grouped as follows: lean (BMI <25; mean BMI 22.2 ± 1.79, N = 5), and overweight (BMI >30; mean BMI 30.3 ± 2.17, N = 5). When grouped, the lean BMI-ASCs formed a significantly higher percentage of colonies (34.94 ± 1.46) compared to the overweight BMI-ASCs (28.26 ± 1.78); p < 0.05 (Figure 2b). Representative photomicrographs of CFUs are shown in Figure 2c. Annexin-V/PI staining and fluorescence-activated cell sorting (FACS) was used to determine whether the compromised growth in higher BMI-ASCs was accompanied by elevated apoptosis. There was no significant difference in apoptosis between lean and overweight BMI-ASCs at 24 and 48 hrs of culture with 2% or 10% FBS (data not shown). The percent early apoptotic cells did not exceed 5.49 ± 1.86%, and the percent late apoptotic cells did not exceed 4.12 ± 0.23% (mean ± SD).

### BMI did not affect late time point adipogenic differentiation in vitro

**Figure 3:**
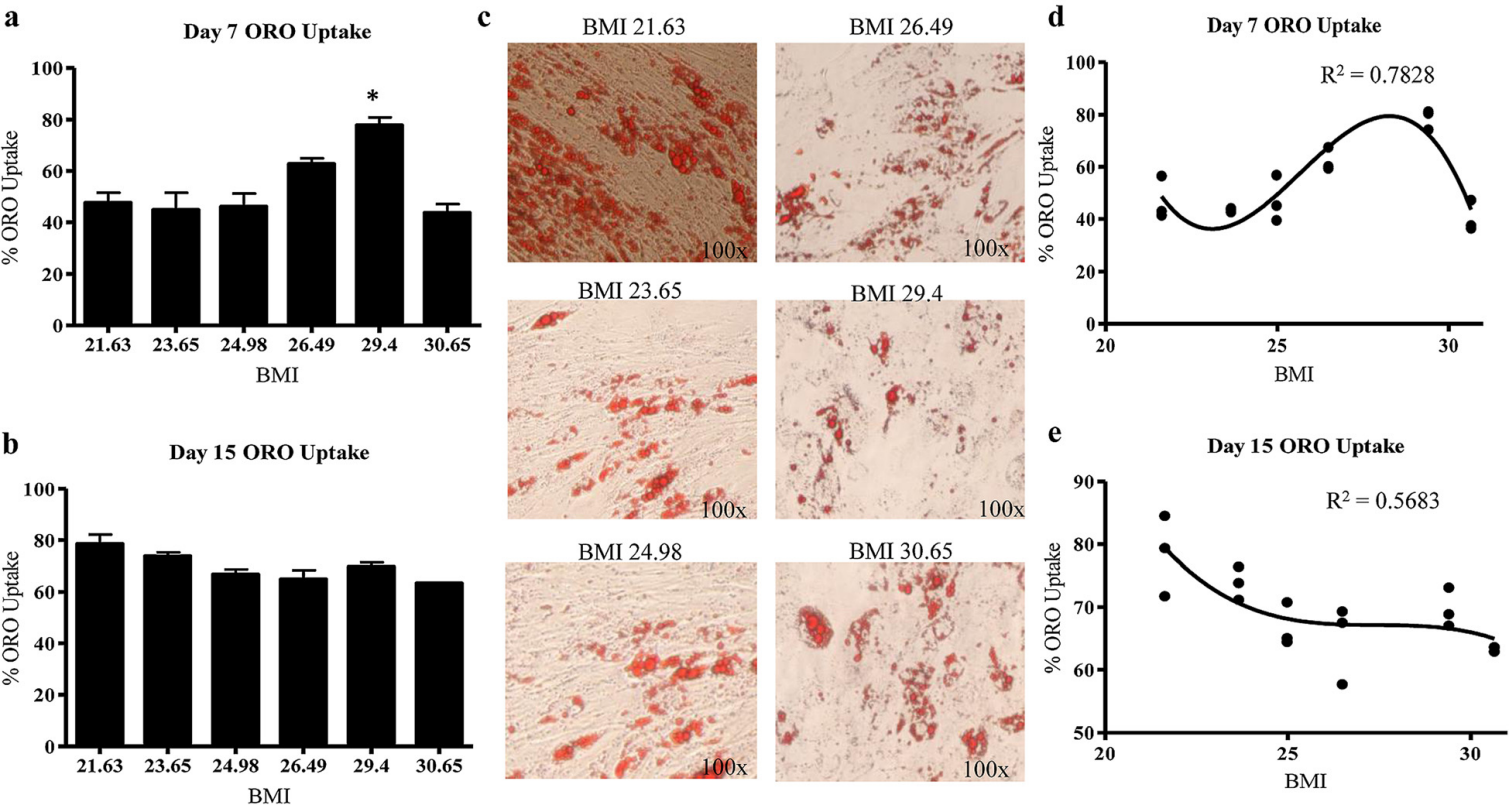
**BMI did not significantly affect adipogenesis potential *in vitro*.** ASC adipogenesis was induced using differentiation induction medium for three days. ASC cultures were then switched to maintenance medium until day 15. Lipid formation was assessed by percent incorporation of oil red-o (ORO) into monolayers cultured in adipocyte differentiation medium for **a**. 7 days, and **b**. 15 days. **c**. Representative micrographs of ORO staining in BMI-ASCs at day 15. **d**, **e**. Regression analyses of differentiation data that was analyzed at days 7 and 15 using least squares ordinary fit. R^2^ value reflected no significant correlation at late adipogenesis timepoints (**e**; Day 15); however, early adipogenesis (**d**; Day 7) R^2^ value reflected a correlation between BMI and adipogenic potential. Values are reported as N ± SE; * p > 0.05, ** p > 0.001.

To investigate the effect of BMI on ASC differentiation, adipogenesis was induced by culturing ASCs in differentiation induction medium for three days followed by culture in maintenance medium until day 15. Differentiation was induced in both 3% and 10% serum. Lipid formation was assessed by percent intracytoplasmic incorporation of Oil Red-O (ORO) into monolayers at days 7 and 15 of adipogenesis. Oil-red-o staining at day 7 revealed a positive correlation between BMI and adipogenesis at early time points (as BMI increased, lipid accumulation increased; Figure 3a). Grouping of BMI-ASCs revealed that overweight BMI-ASCs had significantly higher Oil-Red-O staining (61.40 ± 5.139) compared to the lean BMI-ASCs (46.20 ± 2.70); p = 0.017 at day 7 (data not shown). Staining at day 15 revealed that BMI has no significant effect on ASC adipogenesis during late time points (Figure 3b). To further investigate the correlation between BMI and adipogenesis, nonlinear regression analyses were applied to adipogenesis data from days 7 and 15. R^2^ values reflected a correlation between BMI and adipogenesis at day 7 (R^2^ = 0.78; Figure 3c), and no correlation at day 15 (R^2^ = 0.57; Figure 3d). Representative photomicrographs of ORO staining in BMI-ASCs at day 15 are shown in Figure 3e.

To determine whether cryopreservation was a factor in the observed absence of BMI effects on adipogenic differentiation, we induced adipogenesis in freshly isolated ASCs and cryopreserved ASCs at passage 1 from two of the same donors (mean age 49.5 ± 4.95; BMI 25.6 ± 1.56; Additional file 1: Figure S1a, b). ORO staining (Additional file 1: Figure S1e) demonstrated no significant difference in intracytoplasmic lipid accumulation prior to or following the freeze-thaw process.

### BMI negatively correlated with extracellular matrix mineralization

**Figure 4:**
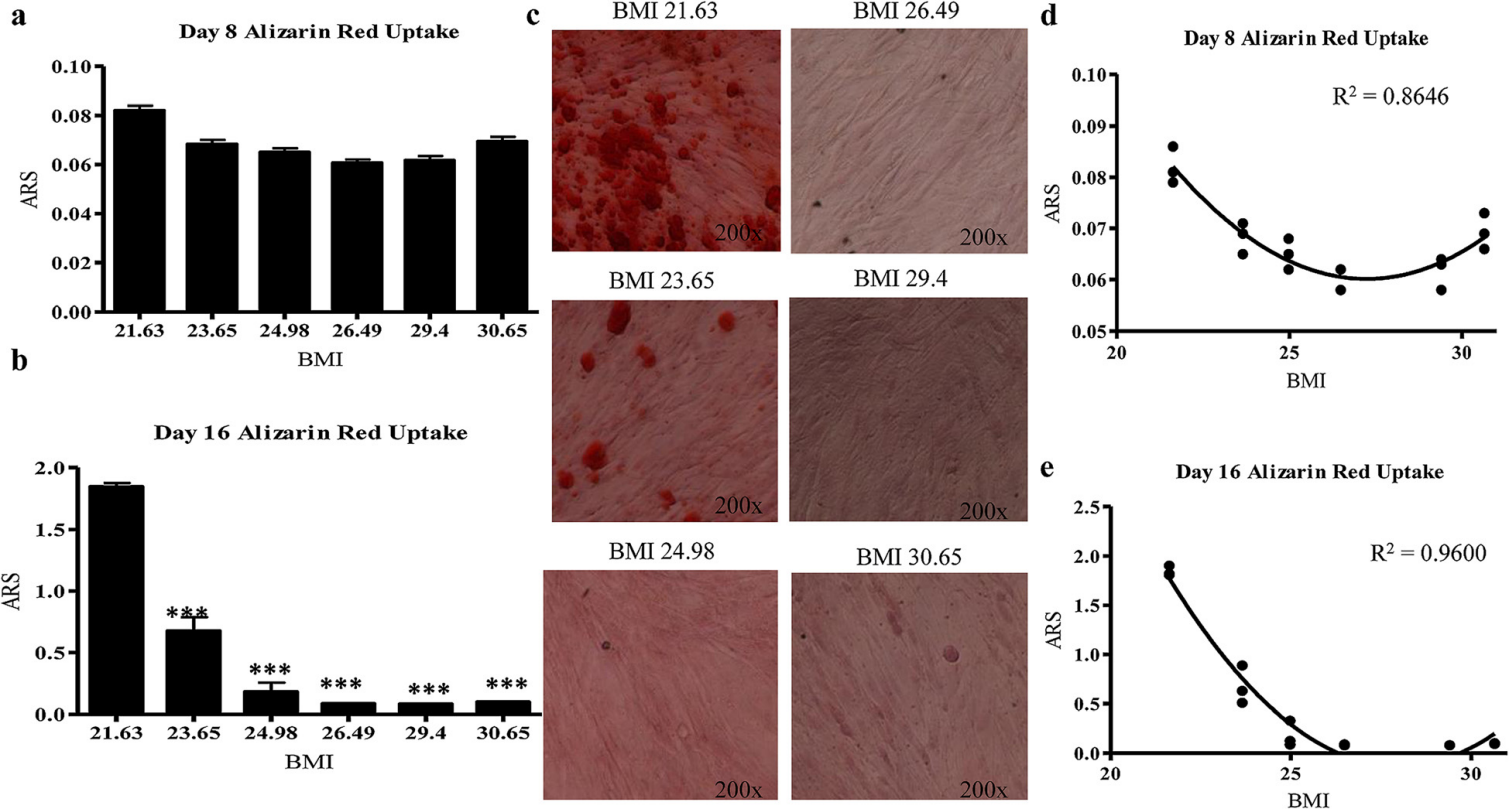
**BMI significantly affected ASC early and late osteogenesis potential *in vitro.*** ASC osteogenesis was induced using a cocktail medium for 16 days. Calcium deposition was assessed by Alizarin red staining (ARS) at **a**. day 8, and **b**. day 16. **c**. Representative micrographs of Alizarin Red Staining (ARS) in BMI-ASCs at day 15. **d**, **e**. Regression analyses of differentiation data that was analyzed at days 8 and 16 using least squares ordinary fit. R^2^ value reflected a strong correlation at both early and late osteogenesis time points. Values reported as N ± SE; * p > 0.05, ** p > 0.001; *** p > 0.0001.

The effects of BMI and percent FBS on ASC osteogenesis at early and late time points was investigated by inducing osteogenesis using an osteogenic cocktail medium for 16 days, as described in the materials and methods. Differentiation was induced in both 3% and 10% serum. Calcium deposition was assessed by Alizarin Red staining (ARS) at days 8 and 16. ARS revealed an inverse correlation (as BMI increased, matrix mineralization decreased) between BMI and ASC osteogenic potential at both time points; this was most pronounced at day 16 (Figures 4a, b). Non linear regression analyses of the ARS data revealed that% FBS had no significant effect on BMI-ASC osteogenic potential, similar to that observed with adipogenesis (data not shown). To evaluate the correlation between BMI and osteogenesis, nonlinear regression analyses were performed on osteogenesis data from days 8 and 16 using the least squares ordinary fit. R^2^ values reflected a strong inverse correlation at both time points (day 8, R^2^ = 0.86; day 16, R^2^ = 0.96; Figures 4c and d, respectively). Representative photomicrographs of ARS in BMI-ASCs at day 16 are shown in Figure 4e. To determine whether cryopreservation was a factor in the observed effects on osteogenic differentiation, we induced osteogenesis in freshly isolated ASCs and cryopreserved ASCs from two of the same donors at passage 1 (mean age 49.5 ± 4.95; BMI 25.6 ± 1.56; Additional file 1: Figure S1c, d). ARS (Additional file 1: Figure S1f) demonstrated no significant difference prior to or following the freeze-thaw process.

### BMI negatively correlated with alkaline phosphatase mRNA expression

**Figure 5:**
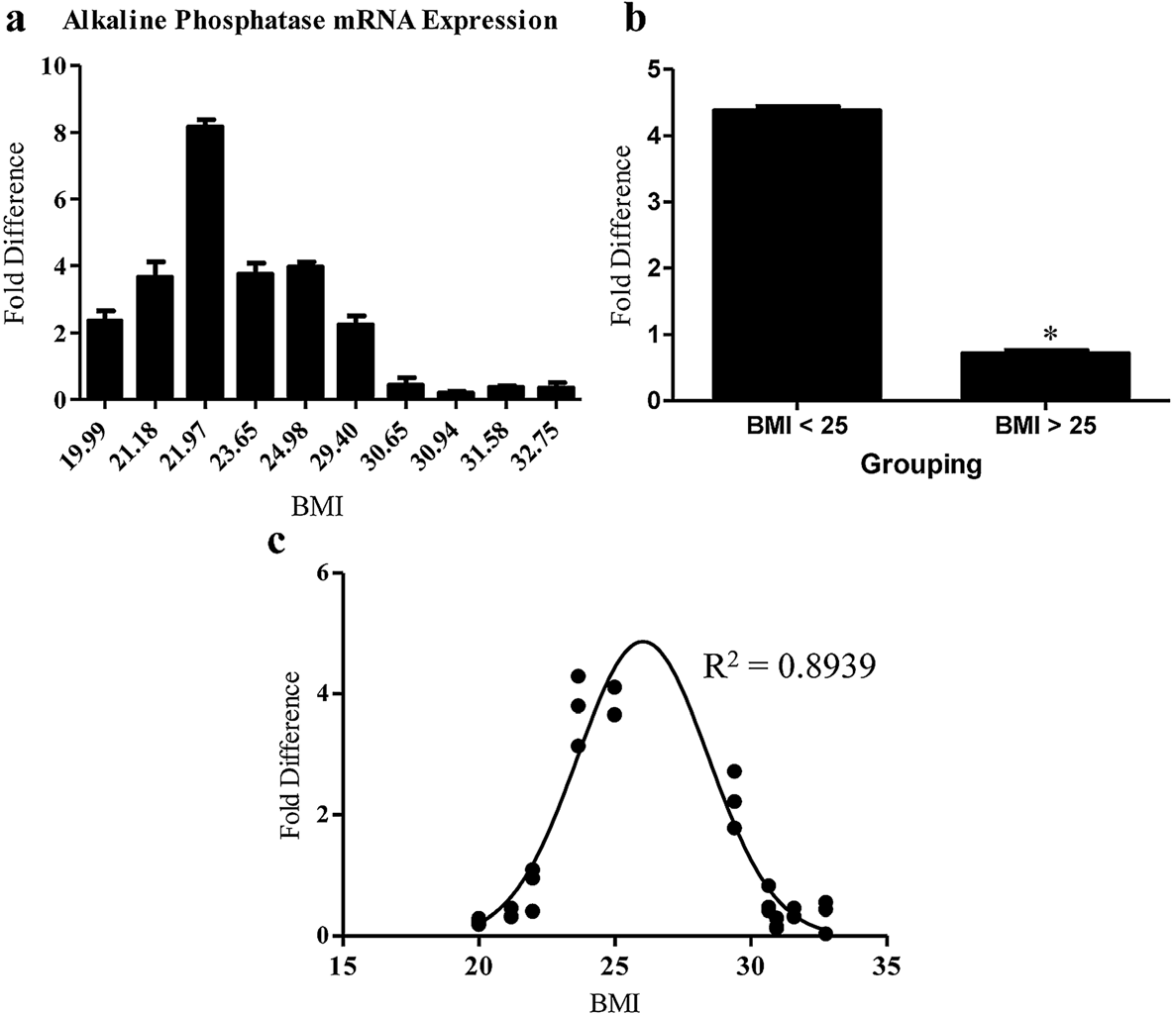
**Expression of alkaline phosphatase mRNA indicated that higher BMI was associated with reduced ASC osteogenesis capacity *in vitro. ***ASC osteogenesis was induced using a cocktail medium for 9 days. RT-PCR was performed using primers and a probe to alkaline phosphatase for a measure of mRNA expression. ΔCT values were normalized to internal GAPDH. **a**. ungrouped averages of triplicates **b**. grouped averages of ‘lean’ (BMI <25 n = 5) and ‘overweight’ (BMI >25; n = 5) BMI-ASCs. **c**. Linear regression analyses of differentiation data that was analyzed at day 9 using least squares ordinary fit. R^2^ value reflected a strong correlation between BMI and osteogenesis. Values reported as N ± SE; * p = 0.017.

To confirm the results of Figure 4 demonstrating a significant effect of BMI on ASC osteogenesis, alkaline phosphatase mRNA expression was measured in additional ASC cultures after induction of osteogenesis. Alkaline phosphatase mRNA results confirmed that when grouped, overweight BMI-ASCs were significantly more compromised in osteogenic differentiation potential (N ± SE: 0.71 ± 0.18) compared to the lean BMI-ASCs (N ± SE: 4.4 ± 0.21; p = 0.017,s Figure 5a,b). Nonlinear regression analyses were performed on osteogenesis RT-PCR data using least squares ordinary fit. The R^2^ value again reflected a strong inverse correlation (R^2^ = 0.89) between BMI and osteogenic potential (Figure 5c).

### BMI did not affect ASC relative cell size and complexity in vitro

Recent studies indicated that mature adipocytes from overweight and obese individuals were smaller in size compared to adipocytes from lean individuals [27]. To determine whether ASCs might exhibit similar relationships between BMI and cell size and complexity, forward-scatter flow cytometry (to approximate cell size), and side- scatter flow cytometry (to approximate cell granularity, e.g. organelles) was performed on BMI-ASCs following culture in 2% or 10% FBS for 24 and 48 hrs. Grouping of forward and side scatter analyses revealed no significant difference in cell size or complexity between lean and overweight BMI-ASCs following 24 hrs of culture in both 2% and 10% serum (Additional file 2: Figure S2).

## Discussion

The potential use of ASCs in tissue engineering and regenerative medicine has been well demonstrated in multiple pre-clinical animal models [24,28]. Although ASCs have been characterized based on immunophenotype, cell yield and differentiation properties [26,29-31], the effects of BMI on ASC functionality have not been fully elucidated. The present study demonstrated that cryopreserved human ASCs from patients with higher BMIs were compromised in colony-forming potential and growth under exposure to low serum concentrations. Overweight BMI-ASCs were also compromised in osteogenic differentiation capacity when compared to lean BMI-ASCs. Cryopreservation was not a factor in the observed differences, as both freshly isolated BMI-ASCs and cryopreserved BMI-ASCs were comparable in both osteogenic and adipogenic differentiation capacities. Moreover, *in vitro* adipogenesis data suggested that early time point intracytoplasmic lipid uptake was positively correlated to BMI. Exposure of ASCs to low serum uncovered differences among BMI-ASCs that were less apparent at higher serum concentrations. Although one individual donor exhibited variation in the trend of BMI and proliferation (e.g. BMI-ASC 29.4), the determination coefficients were sufficient to form conclusions about the correlation between proliferation and BMI.

Overweight BMI-ASC osteogenesis was reduced independent of serum levels. These results were similar to a published report that the BMSC osteogenic alkaline phosphatase response to mechanical strain was inversely correlated to donor BMI [5]. These findings paralleled clinical data demonstrating that obese patients possessed lower relative bone area and bone mass compared to lean individuals, when adjusted for body weight, [15], and this may contribute to an increased risk for fractures [8]. Other clinical studies have demonstrated an inverse correlation between body weight and bone density and peak bone mass. In addition, there is evidence of increased risk of implant failure following total joint knee replacement in obese patients [9-11]. An understanding of the impact of fat metabolism on bone precursors and osteogenic capacity will be necessary to advance ASC use in bone repair applications.

The present data suggests that overweight BMI dependent increase in early adipogenesis may be associated with a decrease in osteogenic capacity. Multiple studies suggest that when ASC fate is committed to the adipogenic pathway, osteogenesis is concomitantly down regulated, consistent with observations in BMSCs [25,32]. This balance is suggested to be partly modulated by the presence and amount of glucocorticoid, the glucocorticoid receptor pathway activity, crosstalk with the Jak/STAT3 pathway, and the subsequent activation of the AP-1 pathway [33,34]. Other studies have suggested that an inverse relationship exists between donor age and ASC osteogenesis [35], and that ASCs from male donors exhibit increased osteogenic potential compared to ASCs from female donors *in vitro*[36]. However, conflicting outcomes were reported in studies comparing donor age and BMSC osteogenic potential *in vitro* with one study demonstrating an inverse correlation [37] whereas other studies reported a positive correlation [38,39].

ASCs from overweight BMI patients (BMI >25) showed an increase in the intracytoplasmic Oil-Red-O staining at early adipogenesis time points, but no significant difference at later time points. Although an increasing trend was observed in early adipogenic potential that correlates to BMI, an individual donor effect may have contributed to the low adipogenesis in donor L070525 (BMI 30.65 kg/m^2^). Genes that are induced during both early and late stage adipogenesis have been extensively described [20,21,33,40-42]. Yu et. al., 2010 [42] performed time-dependent RT-PCR analyses of genes involved in adipogenesis in 4 donors, BMI 23.1 ± 1.4, age 39 ± 6 years. The adipogenic transcription factor C/EBPα, the adipokines adiponectin and leptin, and lipoprotein lipase (LPL) all displayed a time-dependent increase during adipogenesis, with the greatest percent increase occurring within the initial 3-day period; however BMI had no significant effect on the mRNA expression of the representative genes that were upregulated on day 3 of adipogenesis (PPARγ, Adiponectin, LPL, and aP2). This suggests that BMI has no overall effect on adipogenesis *in vitro*, and supports our data demonstrating no correlation between BMI and late adipogenesis. However, Schipper et. al. [43] reported age-dependent differences in ASC adipogenic potential and proliferative capacity *in vitro*. Further investigation is needed relating the impact of age and BMI on early and late ASC functionality.

It is widely accepted that hyperplasia follows adipocyte hypertrophy in patients during fat mass expansion which requires a gradual replenishment of the pool of adipocytes in obese individuals [44,45]. One study proposes that BMSCs are recruited and give rise to bone, muscle, and both white and brown adipose tissues in response to the appropriate developmental cues *in vivo*[46]. However, the ability of ASCs to differentiate into terminally differentiated cells of both white and brown adipose tissues adds further complexity to the interesting dynamic. The formation of newer, small mature adipocytes is thought to occur via differentiation of preadipocytes; however whether the preadipocyte originates from the fat mass, or from the recruitment of the circulating bone-marrow progenitor cells [47] remains to be determined. In this regard, it should be noted that Koh et. al. [28] demonstrated that BMSCs in bone marrow transplant mice became resident as phagocytic macrophages in adipose tissues and resembled trans-differentiated adipocytes, but did not express adipocyte markers.

The present study used cryopreserved ASCs that were passaged *in vitro*, not freshly isolated ASCs. Several studies have investigated the cryopreservation characteristics of ASCs and examined different methods of cryopreservation on ASC biology *in vitro*[41,48-50]. Thirumala et al., 2010 [22,51], showed that the post-thaw viability of ASCs differed significantly based on the method of freezing and rate of thaw, and determined that using an ethanol enclosed jacketed container, with 10% DMSO and 80% FBS in the cryopreservation medium, yielded the highest post-thaw viability. Other studies have examined the effects of various cryoprotective agents on cell viability, proliferation, and osteo- and adipogenic differentiation [47-49]. These studies indicated that proliferation and osteo- and adipogenic differentiation of ASCs could be maintained *in vitro* post-thaw. Our studies also demonstrated that cryopreservation did not significantly affect ASC adipogenic or osteogenic potential (Additional file 1: Figure S1, panels a-f).

Reports on the proliferation index of freshly plated ASCs versus cryopreserved ASCs are conflicting. James et. al., 2011 [39] compared ASCs freshly harvested from lipoaspirate to cryopreserved ASCs, and measured growth using Trypan blue dye exclusion assay. Their results demonstrated cryopreservation significantly affected *in vitro* proliferative capacity. However, studies by Deng et. al., 2008 [52], and Gonda et. al., 2008 [53] suggest that ASC proliferative capacity following cryopreservation is maintained. Calculations of ASC population doubling times in our studies coincide with reports by Deng et. al., 2008, and Gonda et. al., 2008 [52,53], as there was no significant difference observed between the doubling times of freshly isolated ASCs and cryopreserved ASCs (Additional file 1: Figure S1, panel g).

James et. al., 2011 [39] also compared ASCs freshly harvested from lipoaspirate to cryopreserved ASCs, and measured osteogenic differentiation (alkaline phosphatase staining and qRT-PCR), and adipogenic differentiation (alizarin red staining and qRT-PCR). The results demonstrated cryopreservation significantly affected osteogenic differentiation, both *in vitro* and *in vivo*. The use of recombinant proteins such as IGF and BMP, however, were used to mitigate the deleterious effects of the freeze–thaw process on osteogenic potential. It should be noted that BMI was not a tested factor, and no cytokines or signaling factors were added to their differentiation medium. A handful of cytokines and other factors are known to stimulate osteogenic differentiation in ASCs, including vitamin D, which was added in the differentiation medium used in the present study. In addition, the strong determination coefficients derived from performing the nonlinear regression analyses of the proliferation (r = 0.86-0.9) and osteogenic differentiation studies (day 8, r = 0.86; day 16, r = 0.96) suggest donor metabolic status may also have an effect on ASC functionality.

*In vivo*, near complete wound healing was observed in mice with calvarial defects that were engrafted with fresh human ASCs as compared to groups engrafted with cryopreserved ASCs which exhibited reduced healing [39]. Although freshly isolated ASCs may exhibit beneficial properties for wound healing and other applications, the majority of applications for ASCs will likely use cryopreserved ASCs, therefore it is important to understand the characteristics and limitations of cryopreserved ASCs. Future studies comparing the functionality of freshly isolated ASCs to cryopreserved ASCs within different BMI groupings are needed to determine whether the observed differences exist in freshly isolated cells.

Other possible contributing factors to the outcomes of this study are patient clinical characteristics that were unavailable from patient records, such as donor hormonal status, smoking and other undisclosed patient parameters. Regression analyses were conducted to determine whether a correlation exists between age, passage and the measured endpoints within the study. All R^2^ values were less than 0.2 and as such reflect no correlation between the known factors and the measured endpoints.

## Conclusions

While ASCs are proving to be promising candidates for many applications in regenerative medicine and tissue engineering, understanding the phenotypic characteristics of these cells is essential to ensure product consistency and suitability towards specific applications. The present study demonstrated that in comparison to ASCs from lean individuals, ASCs that originate from individuals with higher BMIs had comparable adipocyte differentiation but reduced proliferation and osteogenic potential. Thus, ASCs derived from donors with overweight BMI may be less than optimal for applications that involve usage in biocompatible scaffolds for bone grafting.

## Methods

### Materials

All chemicals were purchased from Sigma-Aldrich (St. Louis, MO) or Fisher Scientific (Norcross, GA) unless otherwise specified.

### Donor demographics

All tissue was obtained from the subcutaneous abdominal adipose tissue region of female, Caucasian patients. The tissue was acquired from elective procedures in local plastic surgery offices, with the patient’s informed consent as approved by the Pennington Biomedical Research Center Institution Review Board. The primary cultures were prepared as described in Dubois et al., 2008 [54]. The tissues used were from 11 female donors of ages between 28 and 61 with a mean ± SD of 41.5 ± 8.61 years. The patients displayed a mean body mass index (kg/m^2^) (±SD) of 26.3 ± 4.62 (summarized in Table 1).

### Isolation, collection, and culture of human ASCs

Both fresh and cryopreserved human ASCs were obtained from the Pennington Biomedical Research Center using the protocol described by Gimble et. al [21,40]. Briefly, liposuction tissues were transported to Pennington laboratory in saline solution within 2 h post-surgery. The tissue was washed at least three times with two volumes of Phosphate Buffered Saline (PBS) to remove blood. The tissue was then digested with one volume of PBS supplemented with 0.1% collagenase type I (Worthington Biochemicals, Brunswich NJ), 1% bovine serum albumin, and 2 mM CaCl_2_ for 60 min at 37°C with intermittent shaking. The floating adipocytes were separated from the stromal-vascular fraction (SVF) by centrifugation (300 × g) for 5 mins at room temperature. The supernatant, containing mature adipocytes was aspirated and discarded and the remaining pellet was identified as the SVF. The SVF cells were suspended and plated immediately in T225 flasks in ASC culture medium (DMEM/F-12 Ham’s, 10% FBS [Hyclone, Logan, UT, http://www.hyclone.com], 100 U penicillin/ 100 g streptomycin/0.25 g fungizone) at a density of 0.156 ml of tissue digest/sq cm of surface area for expansion and culture. This initial passage of the primary cell culture was referred to as passage 0 (P0). For cultivation of fresh ASCs for experiments, P0 ASCs were subjected to trypsinization with 5 mL 0.25% trypsin (Life Technologies, Grand Isle, NY) for 5 minutes. Trypsin digestion was stopped by the addition of an equal amount of ASC culture medium. P0 ASCs were then counted using trypan blue dye exclusion, and re-plated at the specified cell density required for each experiment, as described.

For cryopreservation, the ASCs were resuspended in cryopreservation medium (10% dimethylsulfoxide, 10% Dulbecco’s modified Eagle’s medium [DMEM]/F-12 Ham’s, 80% fetal bovine serum [FBS]), frozen at 80°C in an ethanol-jacketed closed container, and subsequently stored in liquid nitrogen prior to thawing for individual assays. The cells were then replated and expanded in cell factories in the Adult Stem Cell Core at Tulane University. Patient donor information (donor number, body mass index, gender, and age) are shown in Table 1 and were used to categorize ASCs as cells isolated from individuals having either lean or overweight body mass indices (BMI-ASCs). Following expansion, freezing and thawing, cryopreserved P0 ASCs were counted using trypan blue dye exclusion, directly plated and used at passage 1 for all experiments, including the comparison between fresh and frozen cells.

### Cell proliferation assays

Cell growth was measured by the MTT (3-(4, 5-dimethylthiazol-2-yl)-2, 5-diphenyltetrazolium bromide) assay and CyQUANT proliferation assays. For the CyQUANT assay, cells from each BMI were seeded in triplicate in ASC culture medium into 24 multi-well plates (Falcon, BD Biosciences, San Jose, CA) at a density of 2.5×10^3^ cells/cm^2^. Following 24 and 48 hrs of culturing in ASC culture medium supplemented with fetal bovine serum ranges of 0% to 10%, medium was removed from the plates, and the monolayers were rinsed with cold PBS. Plates were then frozen at −80°C overnight. The cells were thawed at room temperature and 200 μl of CyQUANT GR dye/cell lysis buffer (included in the CyQUANT kit, Invitrogen, Eugene, OR, USA) was added to each well. The fluorescence was measured using a Fluostar Optima microplate reader (Fluostar Optima, BMG Labtech; Durham, NC). The excitation maximum was 485 nm, and the emission maximum was 530 nm.

For the MTT assay, cells from each BMI were seeded in triplicate in ASC culture medium into 24 multi-well plates (Falcon, BD Biosciences, San Jose, CA) at a density of 2.5×10^3^ cells/cm^2^. ASC culture medium supplemented with fetal bovine serum ranges of 0% to 10% was replaced with fresh ASC culture medium, and 10 μl of 12 mM MTT solution (Invitrogen, Eugene, OR, USA) was added to each well of the 24 multi-well triplicates. The plates were incubated for 4 h at 37°C. MTT formazan crystals were then solubilized by adding 150 μ1 100% dimethylsulfoxide (DMSO) to each well. Plates were then agitated on a plate shaker for 5 min., following which spectrophotometric absorbance at 540 nm was immediately determined using a scanning multi-well spectrophotometer (Fluostar Optima, BMG Labtech; Durham, NC). At least three independent sets of experiments were performed for each treatment.

### Cell viability assessment

To quantify low serum exposure-induced apoptosis, a well-established annexin-V/ propidium iodide (PI) apoptosis staining was performed, and was evaluated by flow cytometry. The control consisted of ASCs treated in fresh ASC culture medium as previously defined (cells were seeded in triplicate into 24 multi-well plates at a density of 2.5×10^3^ cells/cm^2^). Briefly, after 24 hr and 48 hr culture with 2% and 10% serum, both floating and attached cells were pooled, harvested by trypsinization (0.25% trypsin), washed in 10 mL of culture medium and resuspended in 100 μL of 1× annexin-binding buffer (included in annexin V-FITC/PI kit). Cells suspended in a volume of 100 μL were mixed with 8 μL of annexin-V-FITC and 8 μL of 100 μg/ mL propidium iodide (PI) and incubated in the dark at room temperature for 15 min. Apoptotic analyses for ASCs were performed on a fluorescence-activated cell sorter (FACS) flow cytometer (BD Biosciences, San Jose, CA) utilizing 488-nm laser excitation and fluorescence emission at 530 nm and >575 nm.

Apoptosis was characterized by phosphatidylserine (PS) translocation from the inner leaflet to the outer leaflet of the lipid bilayer, while the cell membrane remains intact. Annexin V-positive cells correspond to cells that have experienced PS translocation. PI staining of the cells indicates that the integrity of the cell membrane has been compromised and is used to distinguish living and early apoptotic cells from necrotic cells. Quadrant analysis was performed on the fluorescence dot plot to quantify the percentage of live, necrotic, and apoptotic cell populations, and reported in bar graph form.

### Colony-formation units assay

100 ASCs in 1 ml ASC culture media were plated in triplicates in 6 well plates (10.4 cells/cm^2^) and cultured in a 37°C incubator with humidified 5% CO_2_. Following 14 days of culture, the media was removed, and washed 3 times with 1 mL PBS. 3.0% crystal violet (Invitrogen) in 100% methanol was added and the plates were incubated for 10 minutes at room temperature. The plates were then gently flushed with dH_2_O for 15 min. or until the background was clear. The plates with the stained colonies were examined under an inverted microscope and the number of colonies that were 2 mm diameter or larger were counted using a VersDoc Imaging system (Bio-Rad Laboratories, Hercules, CA). The number of colonies per plate divided by the cells plated × 100 was determined as the “% CFU”.

### Adipogenic differentiation

Adipogenic differentiation of ASCs was performed over a 12 day period as previously described [20]. Briefly, ASCs grown in ASC culture media until the culture reached 90-95% confluency. ASCs were then trypsinized and plated in 24-well plates in ASC culture media at 3×10^4^ cells/cm^2^ for 24 hrs to allow attachment. On day 1 (24 hours after plating), the medium was removed and cells were incubated for three days in adipogenic differentiation medium (Dulbecco’s modified Eagle’s-Ham’s F-12 medium supplemented with 3% or 10% fetal bovine serum, 15 mM HEPES (pH 7.4), biotin (33 μM), pantothenate (17 μM, Sigma), human recombinant insulin (100 nM, Boehringer Mannheim), dexamethasone (1 μM), 1-methyl-3-isobutylxanthine (IBMX; 0.25 mM), and rosiglitazone (1 μM). For the remaining 9 days of the adipocyte differentiation maintenance period, the medium was removed every 3 days and replaced with the same medium that did not contain IBMX and rosiglitazone (maintenance medium).

### Intracytoplasmic lipid quantification

Lipid formation was assessed by incorporation of Oil-Red-O (ORO) (Sigma-Aldrich) into monolayers of ASCs cultured in adipocyte differentiation medium for 12 days. Quantitation of ORO incorporation was performed as previously described [20]. Briefly, 0.5% (w/v) oil red-o (ORO) (Sigma-Aldrich) was prepared in ethanol. 3 parts ORO and 2 parts PBS were then mixed to make a working solution. Monolayers of ASCs cultured in 12-well plates were rinsed 3 times with PBS and subsequently fixed in 10% (v/v) formalin (Sigma-Aldrich) for 15 minutes. The monolayers were then rinsed 3 times with PBS and then incubated in ORO working solution for 45 minutes at room temperature. Following aspiration of unincorporated ORO, monolayers were rinsed 4 times with PBS. Stained monolayers were visualized with phase contrast microscopy (Eclipse 800, Nikon; Tokyo, Japan). Incorporated ORO was extracted by incubating stained monolayers in 100% isopropanol for 10 minutes. The absorbance at 510 nm of each aliquot was then measured using a 96 well plate reader (Fluostar Optima, BMG Labtech).

### Osteogenic differentiation

Osteogenic differentiation of ASCs was performed over a 16 day period as previously described [20]. Briefly, ASCs cultured in ASC culture media until cells reached 90-95% confluency. ASCs were then trypsinized and plated in 24-well plates in ASC culture media at 3×10^4^ cells/cm^2^ for 24 hrs to allow attachment. On day 1 (24 hrs after replating), the medium was changed to BGJb medium (Fitton-Jackson Modification) supplemented with 10% fetal bovine serum, 100 μg penicillin streptomycin/mL, 10 nM dexamethasone, 10 mM b-glycerolphosphate and 50 μg/mL ascorbate-2-phosphate, and 10 nM 1,25-vitamin D3 (osteogenic medium). The cells were induced towards osteogenesis in this medium for approximately 14 days and the osteogenic medium was replaced every 2–3 days.

### Mineralization quantification

40 mM Alizarin Red stain (ARS) (Sigma-Aldrich) was prepared in dH_2_O pH 4.1. ASC monolayers cultured in the 24-well plates were rinsed 3 times with Phosphate Buffered Solution (PBS) and fixed in 10% (v/v) buffered neutral formalin (Sigma-Aldrich) for 15 minutes. The monolayers were then rinsed 3 times with dH_2_O and incubated at room temperature in ARS for 20 minutes with gentle shaking. Following aspiration of unincorporated ARS, monolayers were rinsed with dH_2_0 4 times. Stained monolayers were visualized with phase microscopy (Eclipse 800, Nikon; Tokyo, Japan). Quantitation of ARS incorporation was performed with cetylpyridinium chloride monohydrate (CPC) (Sigma-Aldrich) extraction. Briefly, 10% (w/v) CPC buffer was prepared in Na_2_P0_4_ (pH 7.0). Stained monolayers were incubated in 1 ml of CPC buffer for 45 minutes. 200 μl aliquots of the extracted dye were then transferred to 96 well plates. The absorbance at 550 nm wavelength of each aliquot was then measured using a 96 well plate reader (Fluostar Optima, BMG Labtech; Durham, NC).

### RNA isolation and reverse transcriptase polymerase chain reaction for alkaline phosphatase

Human ASCs were cultured under control conditions or were induced to undergo osteogenic differentiation for 9 days. Total RNA was isolated from the cells using Trizol (Molecular Research Center, Cincinnati, OH). One-step reverse transcriptase-polymerase chain reactions were performed with 200 ng of total RNA using iScript One-Step RT-PCR Kit for Probes (Bio-Rad Laboratories, Hercules, CA). 25 μl 2x RT-PCR reaction mix containing 0.5 mM of each dNTP (dATP, dCTP, dGTP, dTTP), magnesium ions, and iTaq DNA polymerase was added in a Master Mix with 500 nM forward and reverse primers and 250 nM probe for the amino terminal region of tissue non-specific alkaline phosphatase found in liver/bone/kidney (forward start site, 381: 5′-TCGCCTACCAGCTCATGCATAACA-3′; reverse start site, 509: 5′-TGAAGCTCTTCCAGGTGTCAACGA-3′; probe start site, 450: 5′-/56-FAM/TCAGGGACATTGACGTGATCATGGG/3BHQ_1/3′) or GAPDH (forward start site, 117: 5′-TCGACAGTCAGCCGCATCTTCTTT-3′; reverse start site, 210: 5-ACCAAATCCGTTGACTCCGACCTT-S3′; probe start site, 155: 5′-/56-FAM/AGCCACATCGCTCAGACACCATGGG /3BHQ_1/3′). Complete reaction mix was incubated in a real-time thermal detection system (Bio-Rad Laboratories, Hercules, CA). cDNA synthesis was performed using a 10 min 50°C cycle. iScript reverse transcriptase inactivation was performed using a 5 min 95°C cycle, and PCR cycling and detection were performed using 15 sec 95°C, and 30 sec at 60°C. The number of cycles performed was 40. mRNA expression was normalized to GAPDH control and reported as ΔΔCt values for each donor.

### Statistical analysis

Statistical analysis of the data was performed using Graphpad Prism v5.0 software; the level of *P* value was set at 0.05. The Statistical analyses performed were as follows: 2-way analysis of variances (2-way ANOVAs), followed by Bonferroni’s post tests were used for CyQUANT and MTT proliferation experiments, and differentiation experiments. The Student’s one-sample t-tests were used for colony formation and viability experiments. Non linear regression analyses using least fit ordinary squares were also performed on proliferation, and differentiation, and the determination coefficients (R^2^ values) were given to evaluate the relationship between BMI and proliferation, or differentiation, respectively. The coefficient of determination is such that 0 ≤ R^2^ ≤ 1, and denoted the strength of the association between BMI and proliferation, or BMI and differentiation, where 1 represents the strongest correlation. Results were reported as a positive or inverse correlation. A positive correlation reflects an increase in growth, differentiation potential, or size as BMI increases. A negative correlation reflects a decrease in growth, differentiation potential, or size as BMI increases.

## Abbreviations

BMI: Body mass index; ASC: Adipose tissue-derived stem cell; ORO: Oil-red-O; ARS: Alizarin red staining; FBS: Fetal bovine serum; BMSC: Bone marrow-derived stem cell; CFU: Colony-forming unit; FACs: Fluorescence-activated cell sorting.

## Competing interests

The authors declare that they have no competing interests.

## Authors’ contributions

JMG provided BMI-ASCs and was instrumental in planning experiments. JWD expanded BMI-ASCs in the Tulane University Adult Stem Cell Core. HAT performed flow cytometry for forward and side scatter experiments. ESC provided monetary support. TPF performed the experiments and drafted the manuscript. BGR finalized the manuscript. All authors read and approved the final manuscript.

## Authors’ information

TPF is a doctoral student at Tulane University, in the Structural and Cellular Biology department under the Biomedical Sciences Program in New Orleans, LA. JMG is a Professor of Stem Cell Biology at Pennington Biomedical Research Center in Baton Rouge, LA, and an adjunct Professor to Tulane University in New Orleans, LA. JWD is the manager of the Adult Stem Cell Core at Tulane University in New Orleans, LA. HAT is the manager of the Flow Cytometry Laboratory in the Center for Stem Cell Research and Regerative Medicine at Tulane University in New Orleans, LA. ESC is an Associate Professor of Surgery and Director of Plastic Surgery Research at Tulane University in New Orleans, LA. BGR is an Associate Professor and Co-Vice Chair of the department of Structural and Cellular Biology at Tulane University, New Orleans, LA.

## Supplementary Material

Additional file 1: Figure S1 Cryopreservation does not significantly affect BMI-ASC osteogenic and adipogenic potential *in vitro.* BMI-ASCs that were freshly isolated or frozen and thawed, were induced into **a**. **b**. adipogenesis for 15 days or **c**. **d**. osteogenesis for 16 days, as described in the materials and methods section. Oil-red-o staining was used to evaluate adipogenic potential, and Alizarin red stain was used to determine ECM deposition. Quantification of **e**. ORO and **f**. ARS reflected no significant difference in adipogenic or osteogenic potential, respectively, in freshly isolated cells compared to frozen cells. Values reported as N ± SE. Click here for file

Additional file 2: Figure S2 BMI does not significantly affect cell size and complexity *in vitro.* Forward-side scatters were performed on BMI-ASCs post low serum exposure (2% FBS supplementation), and in ASC culture medium (10% FBS) for 24 hrs. **a**. Cell size (Forward scatter) 24 hrs post exposure. **b**. cell complexity (Side scatter) **c**. Representative forward-scatter and side-scatter plots of cellular events from normal BMI-ASCs (BMIs <25; n = 5) and overweight BMI-ASCs (BMIs >25; n = 5) in 10% serum. Values reported as N ± SE. Click here for file
