# Supplementary figures and images for: Body mass index affects proliferation and osteogenic differentiation of human subcutaneous adipose tissue-derived stem cells
Source: BMC Cell Biol. 2013 Aug 7;14:34. doi: 10.1186/1471-2121-14-34 (PMC3750383; doi:10.1186/1471-2121-14-34)

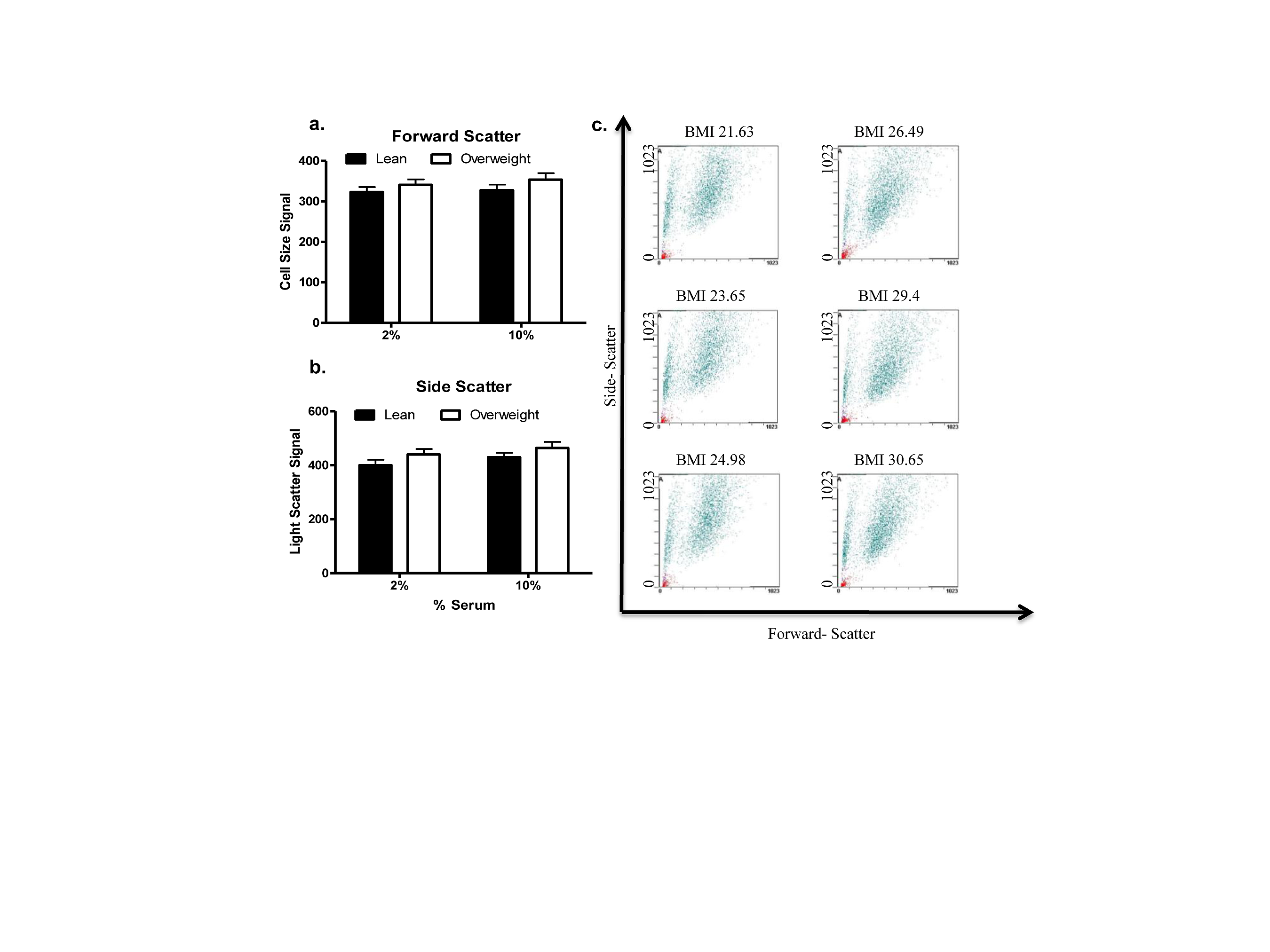

Supplement: Additional file 1: Figure S1 — Cryopreservation does not significantly affect BMI-ASC osteogenic and adipogenic potential in vitro. BMI-ASCs that were freshly isolated or frozen and thawed, were induced into a. b. adipogenesis for 15 days or c. d. osteogenesis for 16 days, as described in the materials and methods section. Oil-red-o staining was used to evaluate adipogenic potential, and Alizarin red stain was used to determine ECM deposition. Quantification of e. ORO and f. ARS reflected no significant difference in adipogenic or osteogenic potential, respectively, in freshly isolated cells compared to frozen cells. Values reported as N ± SE. [file 1471-2121-14-34-S1.tiff]

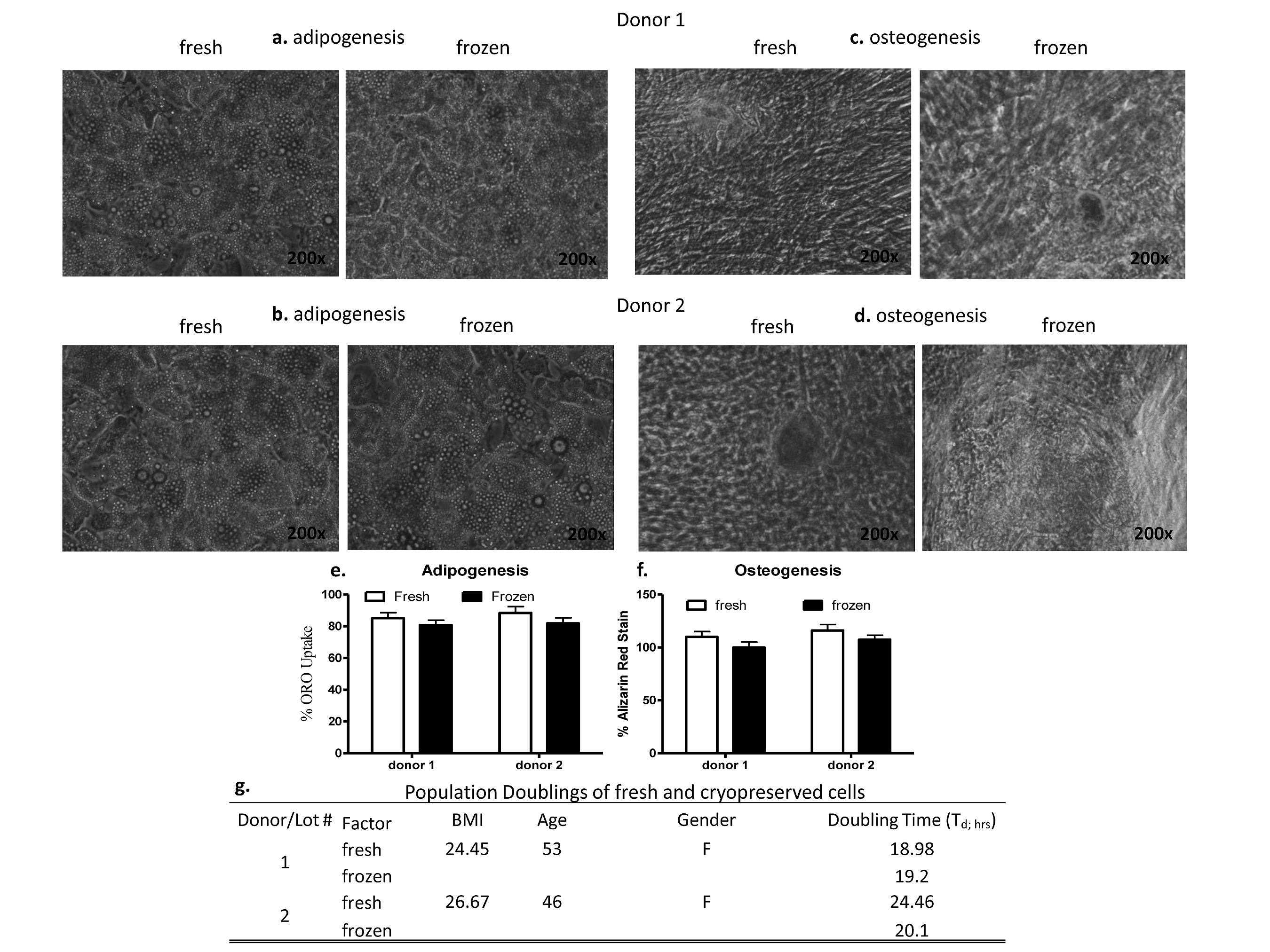

Supplement: Additional file 2: Figure S2 — BMI does not significantly affect cell size and complexity in vitro. Forward-side scatters were performed on BMI-ASCs post low serum exposure (2% FBS supplementation), and in ASC culture medium (10% FBS) for 24 hrs. a. Cell size (Forward scatter) 24 hrs post exposure. b. cell complexity (Side scatter) c. Representative forward-scatter and side-scatter plots of cellular events from normal BMI-ASCs (BMIs <25; n = 5) and overweight BMI-ASCs (BMIs >25; n = 5) in 10% serum. Values reported as N ± SE. [file 1471-2121-14-34-S2.tiff]
